# Case Report: Adult Right-Sided Bochdalek Hernia Complicated by Intrathoracic Bowel Perforation

**DOI:** 10.3389/fsurg.2021.755279

**Published:** 2021-11-19

**Authors:** Jan Philipp Ramspott, Stephan Regenbogen, Tarkan Jäger, Michael Lechner, Franz Mayer, Ana Gabersek, Klaus Emmanuel, Philipp Schredl

**Affiliations:** ^1^Department of Surgery, Paracelsus Medical University Salzburg, University Hospital Salzburg, Salzburg, Austria; ^2^Department of Gynecology and Obstetrics, Münster University Hospital, Münster, Germany; ^3^Department of Trauma Surgery, BG Trauma Center Murnau, Murnau, Germany

**Keywords:** Bochdalek hernia, hernia, repair, mesh, suture, thoracic complication

## Abstract

Right-sided Bochdalek hernia is a mostly congenital condition of the diaphragm caused by a persistence of the pleuroperitoneal cavity and a rare disease in adults. As it often presents as an emergent situation, urgent diagnostics and surgical intervention are essential to reduce morbidity and mortality rates. Choosing the right surgical approach (abdominal, thoracic, or a combination of both) can be very challenging for clinicians. Here, we report a case of a 40-year-old woman, who presented with severe abdominal pain and tachypnoea. Imaging revealed a right-sided Bochdalek hernia. Emergency laparotomy was performed followed by reduction of hernia content, right-sided hemicolectomy, and side-to-side anastomosis from the ileum to the transverse colon due to intestinal ischemia and intrathoracic bowel perforation. The post-operative course was complicated by a pleural empyema. Therefore, the patient underwent thoracotomy. One year after surgical repair the patient had no recurrence. Here, we discuss feasible approaches for the surgical management of complicated Bochdalek hernias.

## Introduction

Diaphragmatic hernias are classified into posterolateral, anterior, or central and occur congenitally or secondarily (post-traumatic) (1). The most common diaphragmatic defect is located posterolateral in the lumbocostal triangle and named after its first describer Vincent Alexander Bochdalek in 1848 (2). As the right pleuroperitoneal canal closes faster than the left one and due to the protective effect of the liver, Bochdalek hernias mainly occur on the left side as an incidental finding in children causing symptoms directly after birth (3, 4). Adult Bochdalek hernias occur much more seldom with an incidence of 0.17% (5). More often women with a mean age of 58 years are affected compared to men and they mostly present with respiratory or gastrointestinal symptoms (6). Hernia formation may be facilitated by former surgical abdominal interventions or increased intraabdominal pressure due to pregnancy or pulmonary diseases (3, 7). In children mortality rates between 42 and 68% are described whereas only rare data about mortality rates in adults can be found (8, 9). Here, we describe the case of an adult non-traumatic rare right-sided Bochdalek hernia with a highly complicated post-operative course due to intrathoracic colon perforation. So far, only 58 cases of right-sided Bochdalek hernias in adults are described (6, 10). Up to date, no large retrospective or prospective studies examining evidence-based diagnostic and treatment strategies were performed due to the rarity of this entity.

## Case Report

**Figure 1:**
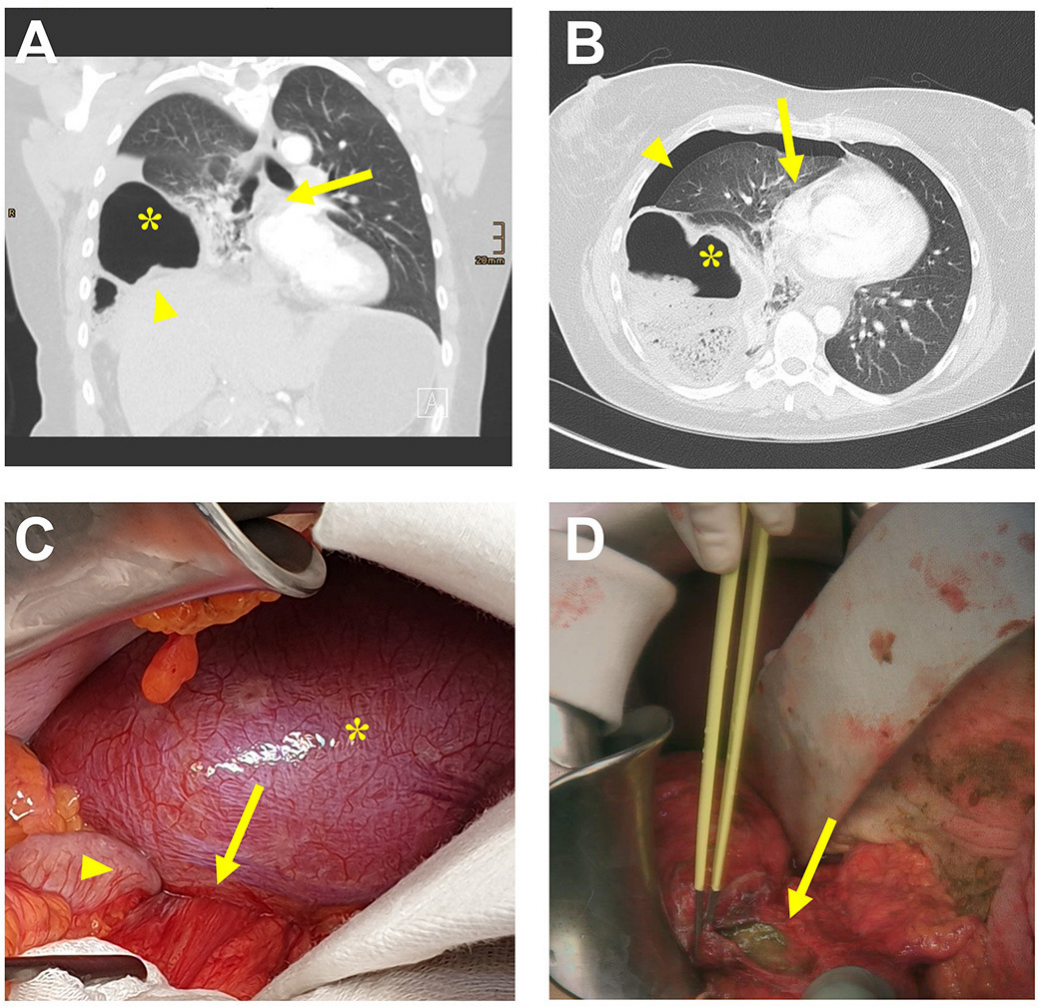
**(A)** Computed tomography (CT) scan (coronal plane) of the chest and upper abdomen showing elevated right hemi-diaphragm (arrowhead), right-sided diaphragmatic herniation of ascending colon (star), and mediastinal shift to the left side (arrow). **(B)** CT scan (axial plane) of the chest showing herniated right-sided ascending colon (star), pleural effusion (arrowhead), and mediastinal shift to the left side (arrow). **(C)** Intra-operative abdominal situs showing the right posterolateral defect (arrow) of the bulged diaphragm (star) with herniation of the ascending colon (arrowhead). **(D)** Intra-operative situs showing the colon perforation (arrow).

A 40-year-old woman with a history of asthma presented to the emergency department with severe upper abdominal pain. The patient's blood pressure was 155/115 mmHg, heart rate was 75 beats/min, and body temperature was 36.4°C. The patient had marked tachypnoea, oxygen saturation was 94% at room air. According to the patient's report, she had regular bowel habits and denied any vomitus, nausea, diarrhea, or constipation. There was no history of previous trauma and no surgical history. Clinical examination revealed a soft abdomen with some slight tenderness and markedly lowered breath sounds over the right lung. Laboratory findings revealed elevated white blood cells (11.14G/L, reference: 3.50–9.80G/L) with negative C-reactive protein. Ultrasound of the abdomen was negative for bilious attack or appendicitis. Pleural ultrasound described pleuritic alterations suggestive of pulmonary infarction. Computed tomography (CT) imaging was added and showed a right-sided Bochdalek hernia with right-sided lung compression, pleural effusion on the right, and mediastinal shift to the left (Figures 1A,B). First, all available findings and the critical situation were discussed with the anxious patient. Imaging results were used to explain the necessary surgical procedure. The patient had sufficient time to ask all relevant questions. Finally, she felt well informed and agreed to the surgical intervention due to lacking alternatives. An emergency median laparotomy was performed under general anesthesia in supine position. A 2 cm right posterolateral diaphragmatic hernial orifice containing an incarcerated and partially necrotic loop of the ascending colon without any intraabdominal signs of infection was detected (Figure 1C). The hernia content was reduced and fecal pleural fluid drained into the abdominal cavity from the chest. Multiple intraabdominal and right-sided thoracic lavages through the hernia defect were performed and the hernial orifice was closed with a non-absorbable 2-0 continuous suture. Right-sided hemicolectomy and a side-to-side anastomosis from the ileum to the transverse colon was necessary due to intestinal ischemia and colon perforation (Figure 1D). A right-sided 24 charierre chest tube was placed and antibiotic treatment with meropenem was started. Histopathological work-up revealed ischemic colon perforation with severe hemorrhagic peritonitis. Initially, the chest tube drained fecal fluid. Drainage reduced over time and the chest tube was removed on the fourth post-operative day. Post-operative pain improved under combined intense intravenous and oral pain medication, which allowed stepwise mobilization.

**Figure 2:**
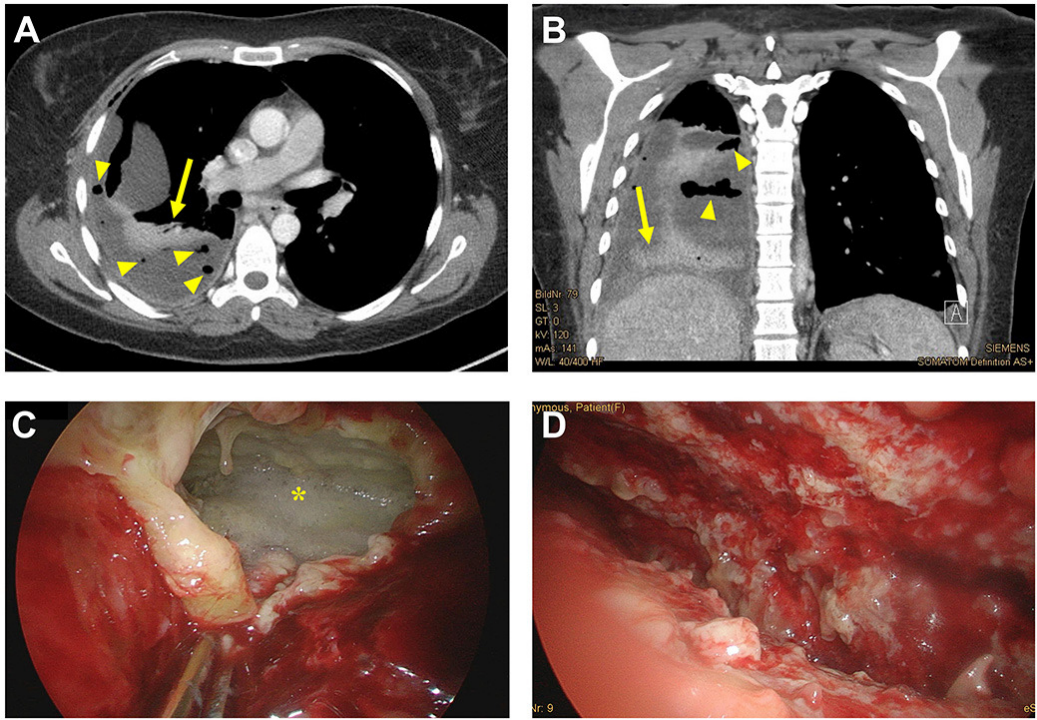
**(A,B)** Computed tomography (CT) scan [axial **(A)** and coronal plane **(B)**] of the chest showing pleural effusion rim enhancement (arrow) with distributed gas (arrowhead) suspicious of pleural empyema. **(C,D)** Intra-operative thoracic situs showing the pleural empyema (star) before **(C)** and after **(D)** intrathoracic lavages were performed.

**Figure 3:**
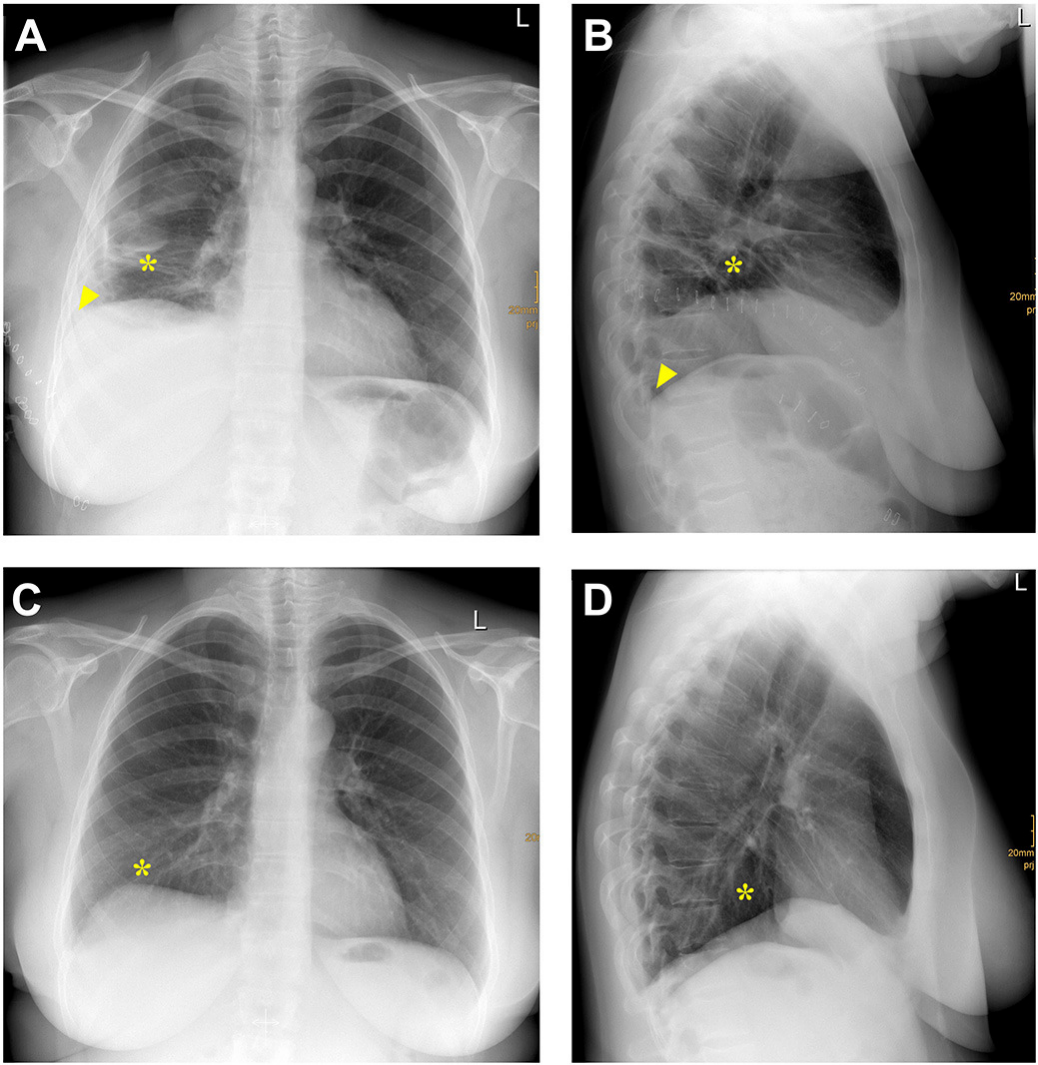
**(A,B)** Posteroanterior **(A)** and lateral **(B)** chest X-ray one month after right-sided Bochdalek hernia repair and empyema evacuation. A right-sided pleural effusion (arrowhead) and opacity due to basal hypoventilation (star) accompanied by a moderate elevation of the right hemidiaphragm without any signs of hernia recurrence can be detected. **(C,D)** Posteroanterior **(C)** and lateral **(D)** chest X-ray one year after right-sided Bochdalek hernia repair and empyema evacuation. No signs of hernia recurrence and only a slight right-basal hypoventilation (star) can be detected.

The further post-operative course was complicated by a right-sided pleural effusion. A thoracentesis was performed, but only a small amount of purulent effusion could be evacuated. Pigtail catheter insertion remained unsuccessful. A thoracic CT scan was added and revealed a pleural effusion rim enhancement with distributed gas suspicious of infected effusion. A 24 charriere chest tube was inserted, which was complicated by multiple pleural adhesions. The chest tube was removed four days later after drainage reduced significantly. As laboratory infection signs remained elevated and the patient became febrile a further thoracic CT scan was performed, which showed increasing rim enhancement now suspicious of pleural empyema (Figures 2A,B). The actual situation was discussed in detail with the patient. Initially, she did not understand why a second surgical intervention was necessary. Once again imaging was presented. The patient gave consent for thoracic surgical intervention on the 13th post-operative day. For explorative video-assisted thoracoscopy (VATS) the patient was intubated with a double lumen endotracheal tube for single lung ventilation and placed in the left lateral decubitus position. A mini thoracotomy was performed at the sixth intercostal space. Due to solid pleuritic formations this approach had to be converted to open thoracotomy following empyema (stage II-III) evacuation (Figures 2C,D) and right-sided pleural decortication. After extensive intrathoracic lavages a 24 charriere right-sided chest tube was placed apical-dorsal and another chest tube in the costodiaphragmatic recess. Antibiotic treatment was switched to levofloxacin and linezolid. The further post-operative course was uneventful and the chest tubes were removed on the fifth and seventh post-operative day, respectively. The early post-operative chest X-ray revealed a small residual right-sided pleural effusion and opacity due to basal hypoventilation accompanied by a moderate elevation of the right hemidiaphragm. No signs of residual empyema or hernia recurrence were detected (Figures 3A,B).

**Figure 4:**
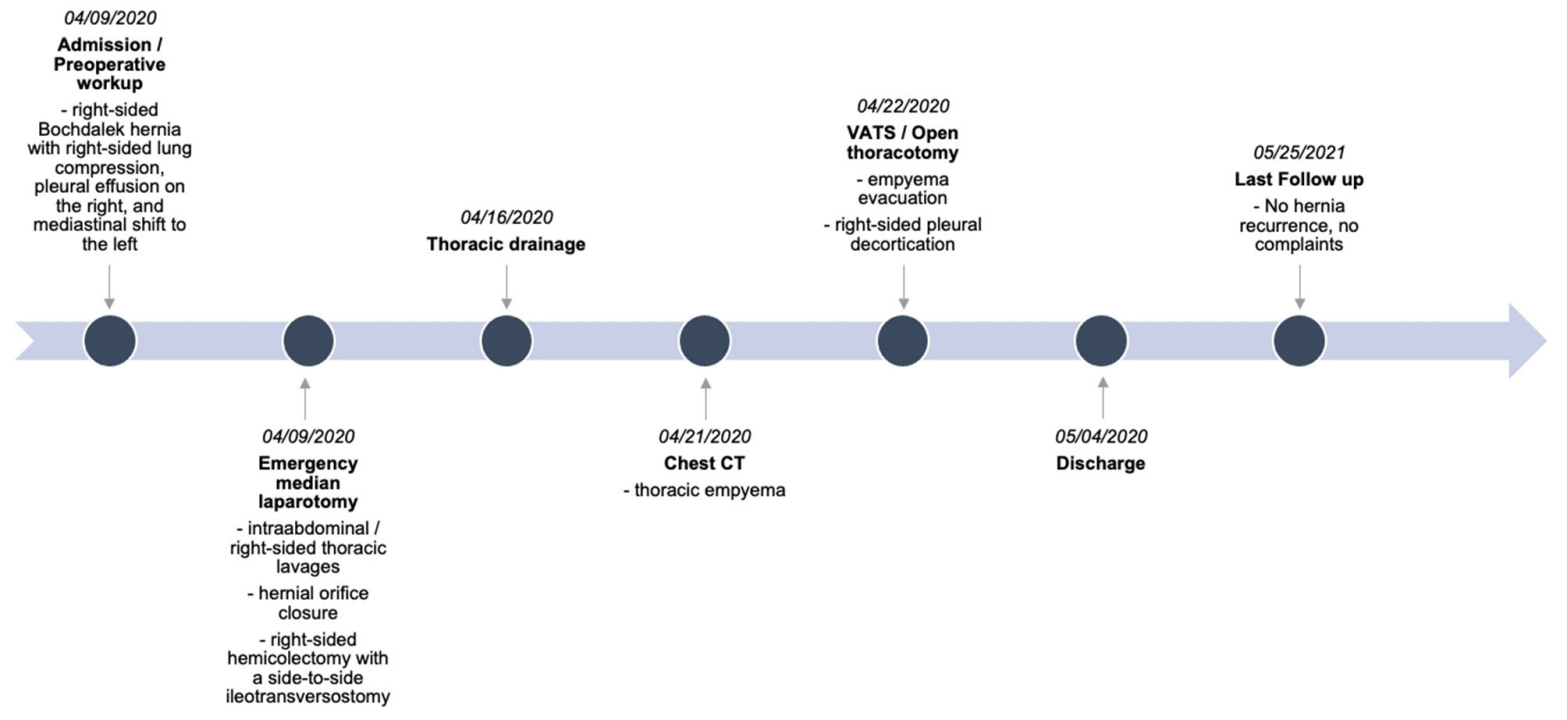
Timeline of pre-operative workup, hospitalization, and follow-up. Computed tomography (CT), video-assisted thoracoscopy (VATS).

The patient was discharged in a stable condition one month after admission. Follow-up clinical examination and chest X-ray one year after initial surgical repair of right-sided Bochdalek hernia did not show any signs of recurrence and only slight right-basal hypoventilation (Figures 3C,D). The summary of the preoperative workup, hospitalization, and follow-up is shown in Figure 4.

## Discussion

The presented case fulfills all clinical characteristics of adult right-sided Bochdalek hernias which were described recently: it is a rare surgical entity often presenting as an emergency situation (6). Besides, women are affected more often than men and the dominating symptoms are abdominal pain and/or dyspnea. In most cases the colon is the dominating herniated organ (6). Here, the patient had a past medical history of asthma that might have increased intraabdominal pressure over time and is known to facilitate hernia development (3, 7).

So far, only three cases of adult right-sided Bochdalek hernias with similar thoracic complications are described (11–13). In one patient intrathoracic abscess formation developed post-operatively after herniated colon resection and diaphragmatic defect repair with absorbable suture by laparotomy was performed. Finally, the abscess resolved under antibiotic treatment and no recurrence was reported after 56 months (11). In contrast, in one further case pre-operative lung empyema did not resolve under multiple antibiotic treatment and drainage. Therefore, the patient underwent a laparotomy with herniated small bowel resection. The hernial orifice was closed by absorbable suture. No outcome was reported (12). In one further patient a fecothorax was detected during the surgical procedure. Necrotic herniated small bowel resection and pleural toilet were performed. Furthermore, a drainage was placed and empyema resolved under intense antibiotic treatment (13).

Similar characteristics of intrathoracic complications and associated treatment strategies can be found between our case and the three cases described in the literature (11–13). Due to fecothorax, a pleural lavage was performed, a drainage was placed, and the antibiotic treatment was continued. But in contrast to the two cases with abscess formation (11) and fecothorax (13), the pleural empyema in the patient described here did not resolve under this therapeutic strategy. Empyema evacuation could only be performed by thoracotomy.

Recently, a systematic review revealed that the adequate diagnostic and surgical procedure of adult right-sided Bochdalek hernias is not completely standardized yet. This might be related to the rarity of this entity (6). Initial clinical examination should focus on both chief complaints dyspnea and abdominal pain (6). Vital signs should be checked continuously. Next, rapid diagnostic imaging (gold standard CT) following surgical repair of each symptomatic and/or complicated hernia presenting with acute incarceration, perforation and/or ileus is necessary to decrease high mortality and morbidity rates. If CT as the gold standard is not available abdominal and chest X-ray, or abdominal and pleural ultrasound should be performed to confirm the diagnosis or to exclude differential diagnoses (6). Any delay of a surgical intervention due to patient's fitness can only be understood as a symptomatic therapy with a poor, likely fatal outcome. Visceral complications like perforation can be managed better by a laparotomy whereas management of thoracic complications like a fecothorax would profit from a thoracic approach. Besides, thoracic diaphragmatic defect repair might be easier especially in right-sided Bochdalek hernias as the liver often masks the hernial orifice. For defect augmentation of the hernia non-absorbable suture as well as non-absorbable mesh in accordance with the general principles of hernia surgery is strongly recommended. In a contaminated setting like intraabdominal or intrathoracic bowel perforation mesh implantation should only be considered in case of hernia recurrence. Any contamination and inflammation must be excluded. As complications of Bochdalek hernias tend to aggravate through secondary complications, thorough exploration of both the abdominal and thoracic cavity must be considered. If a thoracoabdominal approach is necessary, the combination of open and minimally invasive surgery should be preferred. At any time, patient's transfer to a higher-level hospital should be considered depending on patient's fitness, availability of intensive care units, and two-cavity surgical expertise including advanced thoracoscopy (6). If travel distances to a higher-level hospital are too long and extended surgical expertise is missing, damage control surgery should be considered. This includes bowel resection, vacuum-assisted closure (VAC) and intrathoracic/intraabdominal drainage. Afterwards, urgent transfer under antibiotic treatment, intravenous fluids, and pain management to a higher-level hospital for definitive surgical management is mandatory. Otherwise, symptomatic therapy remains the online available option which will lead to a highly complicated outcome.

Considering the complicated post-operative course of the presented case, a simultaneous thoracic approach followed by extensive intrathoracic exploration might have proven beneficial and prevented later empyema development.

Since there is a clearly need for evidence-based diagnostics and treatment strategies and in line with the recently presented current management pathway, regular follow-up visits after right-sided Bochdalek hernia repair are strongly suggested, including imaging and clinical examination up to ten years after surgery (6). Besides, only the reporting of all clinical information and medical work-up of this rare entity in a standardized database will eventually generate more evidence-based knowledge (14).

## Conclusion

In line with other published cases here we report a complicated post-operative course of a non-traumatic right-sided Bochdalek hernia in adulthood. Despite the patient's survival and uneventful abdominal recovery, initially performed extended thoracic management by VATS or thoracotomy might have proven beneficial. In complicated Bochdalek hernias a combination of a thoracic and abdominal approach should be considered to avoid a further complicated post-operative course as this will seriously affect the prognosis. Furthermore, due to its rare incidence only standardized reporting and regular follow up-visits will generate more evidence-based knowledge about optimal diagnostic and surgical intervention of right-sided Bochdalek hernias.

## Data Availability Statement

The original contributions presented in the study are included in the article/supplementary material, further inquiries can be directed to the corresponding author.

## Ethics Statement

Ethical review and approval was not required for the study on human participants in accordance with the local legislation and institutional requirements. The patients/participants provided their written informed consent to participate in this study. Written informed consent was obtained from the individual(s) for the publication of any potentially identifiable images or data included in this article.

## Author Contributions

All authors listed were involved in the patient's care, have made a substantial, direct, and intellectual contribution to the work and approved it for publication.

## Conflict of Interest

The authors declare that the research was conducted in the absence of any commercial or financial relationships that could be construed as a potential conflict of interest.

## Publisher's Note

All claims expressed in this article are solely those of the authors and do not necessarily represent those of their affiliated organizations, or those of the publisher, the editors and the reviewers. Any product that may be evaluated in this article, or claim that may be made by its manufacturer, is not guaranteed or endorsed by the publisher.
